# The Unsuccessful Twiddler: A Case of Twiddler's Syndrome Without Deep Brain Stimulator Lead Breakage

**DOI:** 10.7759/cureus.7786

**Published:** 2020-04-22

**Authors:** Hammad Ghanchi, Taha M Taka, Jacob E Bernstein, Samir Kashyap, Ajay K Ananda

**Affiliations:** 1 Neurosurgery, Riverside University Health System Medical Center, Moreno Valley, USA; 2 Neurosurgery, University of California Riverside, Riverside, USA; 3 Neurosurgery, Kaiser Permanente, Los Angeles, USA

**Keywords:** twiddler's syndrome, parkinson's disease, deep brain stimulator

## Abstract

The authors present the case of a 78-year-old right-handed female with a past medical history of Parkinson's disease, treated with implantation of a left-sided subthalamic nucleus St. Jude Medical Infinity® (Abbott Medical, Austin, TX) deep brain stimulator, who presented with lead-associated discomfort, or “bowstringing”. Further investigation by chest X-ray revealed an extensive case of distal lead coiling. However, it was surprising that, despite the extensive coiling, the lead stayed intact without hardware failure as proven by patient remaining asymptomatic from her Parkinson's disease and intraoperative impedance testing demonstrating normal results. After revision surgery, the patient remained asymptomatic. Due to paucity of cases of this disease in the literature, specific predictive risk factors are not known, but certain patient characteristics may help take precautions.

## Introduction

A deep brain stimulator (DBS) consists of an implantable pulse generator (IPG) that is used as treatment for multiple conditions such as Parkinson’s disease, essential tremor, and other movement disorders. The implantation of a DBS requires placement of the pulse generator into a subcutaneous pocket, most often in the subclavicular space. Twiddler’s syndrome is a known, yet rare patient complication that develops in 1.3% of DBS patients and occurs when the patient turns or twists the implant with eventual lead breakage and failure of hardware [1]. The recurrence of pre-DBS symptoms in patients as well as the presence of pain and discomfort, referred to as “bowstringing”, along the course of the lead should prompt further investigation of the leads and IPG. Examination of the leads through chest X-ray in patients presenting with Twiddler’s syndrome will reveal entanglement, coiling seen as a double-helical pattern, and breakage of the pacing lead [2]. In this report, the authors address a case of Twiddler’s syndrome presenting without lead breakage from an implanted Abbott St. Jude Medical Infinity® DBS System (Abbott Medical, Austin, TX).

## Case presentation

**Figure 1:**
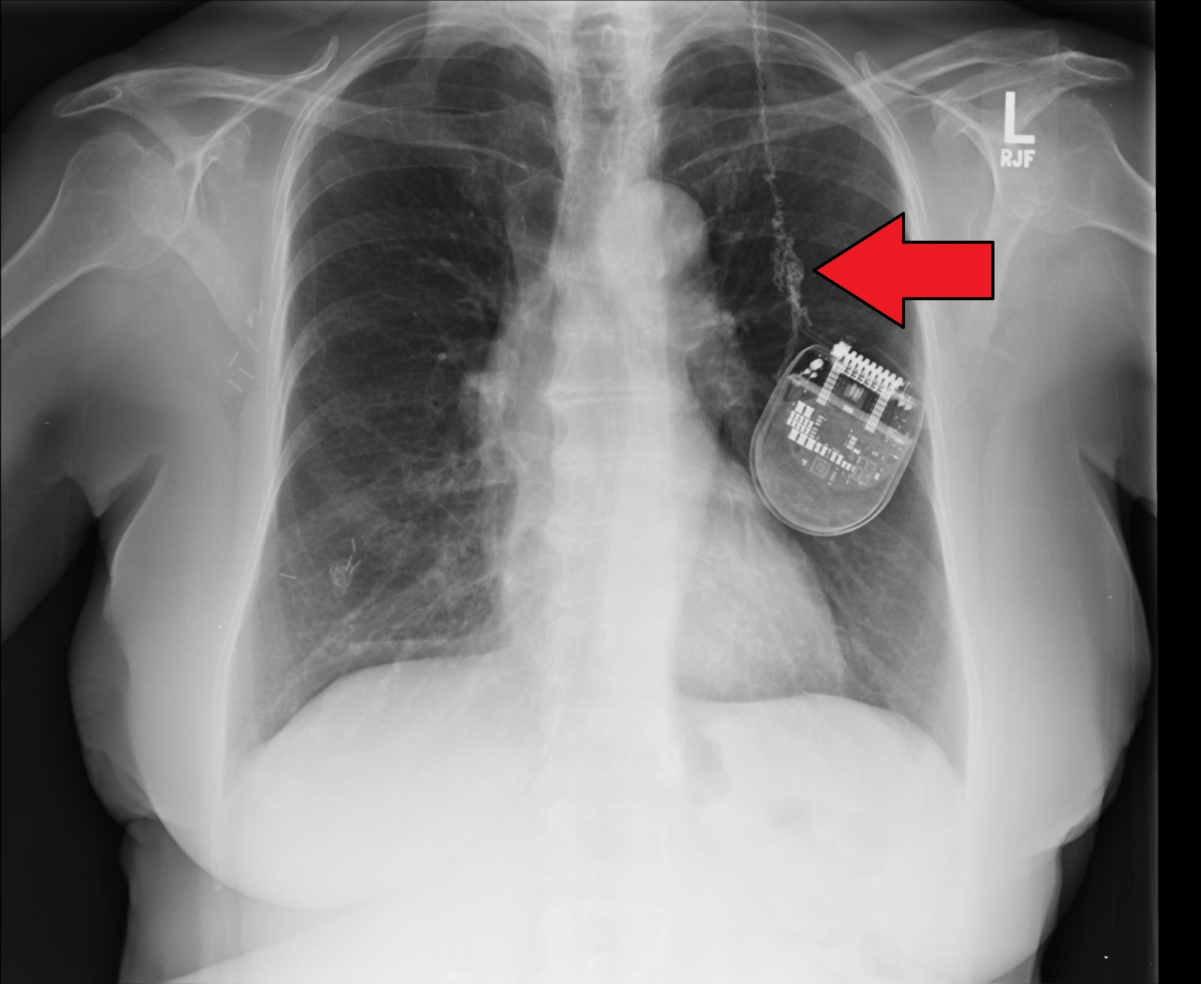
Chest X-ray demonstrating coiled leads on the left

A 76-year-old female with a long-standing history of Parkinson's disease underwent placement of a left-sided subthalamic nucleus St. Jude Medical Infinity® DBS. Postoperatively, the patient had excellent results with her right-sided hemibody symptoms. However, approximately four months after surgery, she presented with complaints of being able to feel the lead pulling in her neck. Chest X-ray revealed multiple coils within the chest cavity indicating that the pulse generator/battery had been flipped many times (Figure 1). The patient denied turning or twisting the battery. Additionally, the patient’s family denied seeing the patient touch her chest to twiddle the battery. The DBS continued to function without any lead breakage or increase in impedance. The patient returned to the clinic multiple times complaining of bowstringing and a decision was made to proceed with open surgery to investigate the coils.

**Figure 2:**
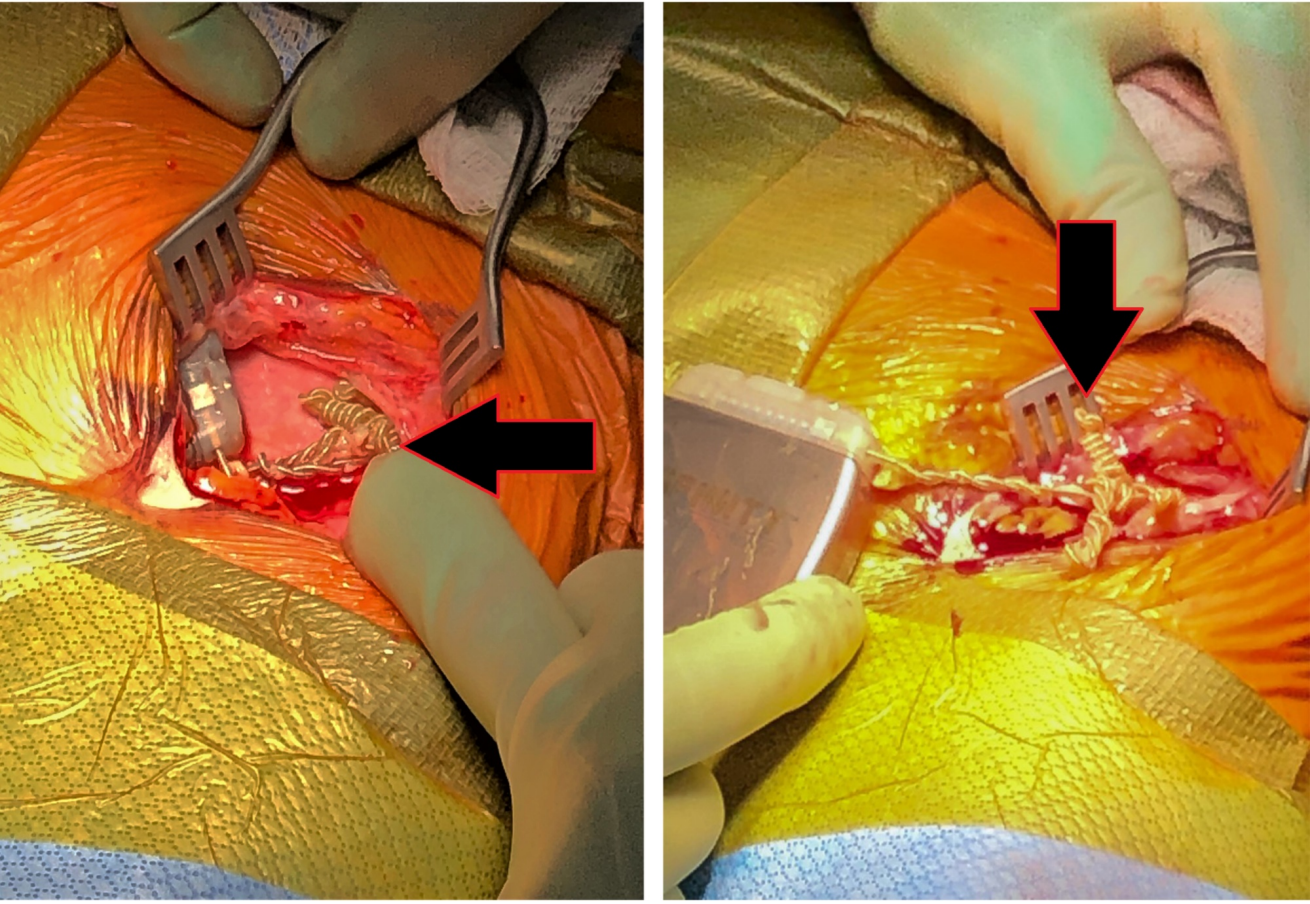
Intraoperative view of the multiple coils noted once the battery was removed

Intraoperatively, the pulse generator/battery was found to be completely intact in the subclavicular pocket. However, the distal portion of the extension lead was found to be twisted into many coils (Figure 2). Impedances were checked once more prior to the removal of the battery from the chest cavity and it still demonstrated a functional DBS circuit. The wires uncoiled without resistance by turning the battery clockwise. A decision was made to replace the extension lead to eliminate the possibility of memory within the lead to re-coil and to prevent any lead breakage that may have occurred from the twisting and coiling of the lead under such tension. The pulse generator/battery was secured to the pectoralis fascia using two 2-0 nonabsorbable monofilament sutures. The subclavicular pocket was investigated, and the pseudocapsule was closed tightly around the IPG to eliminate any potential space.

Postoperatively, the patient recovered well and reported relief of the bowstringing. The patient was shown intraoperative pictures that confirmed that the leads had been coiled. Such twisting, all in the same direction, would have been highly unlikely to occur spontaneously. The patient continued to deny twiddling of her pulse generator/battery. Six months postoperatively, the patient has done well and does not have any recurrent complaints of bowstringing. She continues to deny manipulation of her IPG.

## Discussion

DBS hardware failure from Twiddler’s syndrome is a known and rather recent occurrence in the literature. Twiddler’s syndrome first appeared as a rare yet dangerous complication of cardiac pacemakers, discussed in 1968 by Bayliss as lead fracture and hardware failure occurrences, which may be life-threatening for patients dependent on the machine. As DBS implantations became more common, complications from Twiddler’s syndrome were reported in this patient population as well. The first reporting of Twiddler’s syndrome was in 2007 by Geissinger and Neal in a patient who was treated for debilitating essential tremor and presented later with twisting and fracturing of the leads [3]. 

In DBS patients, Twiddler’s syndrome presents with two characteristic features: failure of the DBS hardware and recurrence of patient’s symptoms prior to DBS placement. However, we present a case where the hardware did not fail and the reason for hardware revision was patient discomfort from the development of bowstringing of the lead in the patient’s neck.

Despite the extensive number of coils present on the radiograph and interoperative findings, St. Jude Infinity® leads did not fracture under stress. Our review of the literature reveals another instance in which the Medtronic Kinetra® (Medtronic, Minneapolis, MN) DBS leads proved resilient against extensive coiling without breakage [4]. From comparison of both leads, the St. Jude Infinity® lead, with a diameter of 1.4 mm, appears more resilient than the Medtronic® DBS leads, with a diameter of 1.27 mm, which may explain its ability to resist the substantial tensile stress. Moreover, in anecdotal experience with handling both systems intraoperatively, the St. Jude Infinity® system seems to be more resistant to intraoperative manipulation and tunneling compared to Medtronic® where the leads or contacts can easily be damaged.

Although the incidence rate of discomfort associated with wire tethering, known as bowstringing, is unknown, its presentation can serve as both an indicator of the underlying complication and a warning sign of further lead malfunction. While the mechanism for bowstringing is not entirely understood, it is thought that the pathogenesis of the bowstringing mechanism is likely due to the scar formation that builds within the course of the lead. In the presence of a foreign agent, the tissue surrounding the lead creates fibrotic capsules that progressively build and contract in a process called “capsular contracture” [5,6]. Additionally, the presence of two parallel leads can increase the fibrotic generation within the lead course due to the increased tension on the subcutaneous channel [7].

Surgical measures can be taken to secure the IPG and prevent its twirling. This is done by fixating the IPG within a tight-fitting subcutaneous pocket using a nonabsorbable silk suture that is passed through the designated IPG hole and fastened to the muscle, fascia, or artificial pouches [8-10]. This process may be inhibited in overweight patients due to the depth of the fascial layer and the overlying fat layer that can impede the postoperative reprogramming. In such cases, an artificial pouch or a tight-fitting pocket is essential [1].

In addition to the intraoperative challenge that is faced with securing the IPG to overweight patients, the presence of relaxed/loose tissue facilitates the rotation of the pacemaker, predisposing obese and elderly patients to the Twiddler’s syndrome [11,12]. Studies also show a predisposition of younger patients, with increased mobility and ability to perform repetitive movements, to have unintended twisting of the leads [12]. Patients with psychiatric disorders, such as dementia, anxiety, obsessive-compulsive tendencies, or paranoia, are also predisposed to the Twiddler’s syndrome; however, due to the rarity of this complication, an incidence rate is difficult to determine [1,13]. Similar to the case we present, it is often noticed that patients deny twisting the device despite the presence of extensive coiling that is very unlikely to have occurred spontaneously. This “unconscious/subconscious manipulation” has been proposed as a phenomenon driven by patients' lack of insight regarding their illness and treatment. Postoperative pain in the place of surgery can trigger an automatic reflex of manipulating the IPG in an effort to relieve the pain, which could be counteracted by the patients' understanding of their treatment and possible complications [13]. For this reason, it is important to educate patients regarding their condition as a preventative measure against twiddling.

At our institution, we commonly perform a single-sided intracranial implantation and subsequently tunnel two extension leads from the parieto-occipital region to the subclavicular pocket to “prewire” the patient for a planned cranial implantation; for most cases, this is a contralateral cranial implantation done a few months to years later, but it can serve to accommodate an ipsilateral implantation if a different target is required for optimal disease control. This avoids opening the subclavicular pocket for the second implantation and also avoids the need for general endotracheal anesthesia as the isolated cranial portion can be done with just local anesthesia. A similar situation occurred in our presented patient who had a single intracranial lead and two extension leads that coiled. For the revision surgery, we implanted a single extension lead to further reduce bowstringing.

Investigation of the lead for Twiddler’s syndrome should be prompted after recurrence of the symptomatology present prior to DBS placement. It should be noted that, similar to the case we present, bowstringing can present alone as a warning sign prior to any recurrence of symptoms. The most effective imaging method for Twiddler’s syndrome is chest X-ray, which will reveal a radio-opaque double-helical pattern and possible further coiling of the lead [14].

## Conclusions

Twiddler’s syndrome in DBS patients usually presents with failure of hardware due to lead breakage. Our report highlights a unique case of Twiddler's syndrome with leads intact and functional, a feature that may be secondary to newer and more resilient leads. Specific risk factors have not been found to be statistically significant given the limited cases; however, factors identified include loose connective tissue in obese or elderly patients, and psychiatric disorders, such as anxiety, dementia, and obsessive-compulsive disorder. Steps to minimize include creation of a tight-fitting subcutaneous pocket with nonabsorbable silk or monofilament sutures along with adequate education of patients regarding their treatment. We also recommend replacement of the extension lead, even if it is not already damaged, to prevent any memory in the lead resulting in recurrence of coils.
